# An interpretable prognostic model for early unfavorable outcomes after bronchiectasis surgery

**DOI:** 10.1186/s12890-026-04508-4

**Published:** 2026-07-16

**Authors:** Yichen Hou, Qibatu Wu, Long Ma, Liwei Zhang, Yanchao Deng

**Affiliations:** State Key Laboratory of Pathogenesis, Prevention and Treatment of High Incidence Diseases in Central Asia, Department of Thoracic Surgery, The First Affiliated Hospital of Xinjiang Medical University, No. 137, South Liyushan Road, Xinshi District, Urumqi, Xinjiang Uygur Autonomous Region 830011 P. R. China

**Keywords:** Bronchiectasis, Lung resection, Prediction model, Penalized regression, Nomogram, QoL-B, Clavien-Dindo classification, Internal validation

## Abstract

**Background:**

Bronchiectasis is a heterogeneous airway disorder characterized by irreversible bronchial dilatation, chronic sputum production, recurrent infections, and acute exacerbations. Although lung resection remains a valuable option for patients with localized, refractory disease despite optimized medical therapy, early postoperative recovery varies substantially among individuals. An interpretable tool for individualized risk stratification remains lacking in clinical practice.

**Methods:**

This retrospective study enrolled patients who underwent bronchiectasis-related lung resection at our center between January 2020 and August 2025 (*n* = 142). The primary endpoint was a composite outcome assessed at approximately 1 month postoperatively. A favorable outcome was defined as the absence of Clavien-Dindo grade II or higher complications in conjunction with a Quality of Life Questionnaire-Bronchiectasis Respiratory Symptoms Scale (QoL-B-RSS) score > 50; all other cases were classified as unfavorable outcomes. Candidate predictors (*n* = 40) were prespecified before model development. Elastic Net-penalized logistic regression was employed as the primary modeling approach, based on which a nomogram was constructed. Nested cross-validation was used for hyperparameter tuning and internally unbiased performance evaluation. Out-of-fold (OOF) predicted probabilities were used to assess discrimination (area under the curve [AUC]), overall accuracy (Brier score), calibration, and clinical utility via decision curve analysis (DCA). A random forest (RF) model, using identical outer-fold partitions, was developed as a direct comparator, and a conventional multivariable logistic regression model was additionally fitted using the predictors retained in the primary model.

**Results:**

The incidence of early unfavorable outcomes was 57.7% (82/142). The final Elastic Net model retained five predictors: pCO₂, left ventricular ejection fraction (LVEF), forced expiratory volume in 1 s as a percentage of predicted (FEV1%pred), maximal voluntary ventilation as a percentage of predicted (MVV%pred), and total Bronchiectasis Severity Index (BSI) score. Internal OOF validation yielded an AUC of 0.820 and a Brier score of 0.173 for the primary model, compared with an AUC of 0.831 and a Brier score of 0.179 for the RF model. Overall, the RF model did not demonstrate a meaningful performance advantage over the primary model. Calibration analysis indicated good agreement between predicted and observed probabilities, and DCA suggested potential net clinical benefit across a range of threshold probabilities.

**Conclusions:**

This internally validated Elastic Net-based nomogram showed good discrimination, acceptable calibration, and potential clinical utility for predicting early unfavorable outcomes after bronchiectasis surgery. Based on routinely available preoperative variables, the model may provide quantitative support for individualized perioperative risk stratification and early follow-up planning. Prospective external validation and clinical impact assessment are warranted to confirm its generalizability and inform future clinical implementation.

**Supplementary Information:**

The online version contains supplementary material available at https://doi.org/10.1186/s12890-026-04508-4.

## Background

Bronchiectasis is a heterogeneous airway disorder characterized by irreversible bronchial dilatation, chronic productive cough, recurrent infection, and acute exacerbations. Daily symptoms and exacerbations not only markedly impair quality of life but also impose a substantial healthcare burden. The latest European Respiratory Society clinical practice guideline highlights reduction of symptom burden, improvement of quality of life, prevention of exacerbations, and delay of disease progression as central management goals, while the British Thoracic Society guideline similarly emphasizes standardized evaluation and management centered on symptom control, infection management, and improved outcomes [1, 2]. Systematic reviews and recent high-quality overviews have highlighted that contemporary bronchiectasis care should adopt an integrated approach addressing symptoms, quality of life, exacerbations, and disease progression [3–5].

For patients with localized refractory disease, recurrent infections, persistent hemoptysis, or inadequate symptom control despite optimized medical therapy, surgical resection remains an important therapeutic option. Existing guidelines and retrospective studies consistently indicate that lung resection is generally safe in appropriately selected patients with localized bronchiectasis and can yield meaningful improvements in symptom control and quality of life [2, 6, 7]. A meta-analysis has further demonstrated low perioperative mortality and an acceptable overall complication rate following lung resection for bronchiectasis [8]. Nevertheless, short-term postoperative recovery remains highly heterogeneous: some patients experience uneventful recovery with substantial symptomatic improvement, whereas others develop clinically significant complications or achieve limited symptomatic benefit. Notably, prior studies on postoperative risk prediction in bronchiectasis have predominantly focused on individual complications, such as postoperative pneumonia; although informative for identifying patients at risk of specific events, such approaches may not adequately reflect overall early postoperative recovery [9].

From the perspective of patients, treatment success in bronchiectasis cannot be sufficiently captured by the mere presence or absence of complications. The Quality of Life Questionnaire-Bronchiectasis (QoL-B) is the most widely used disease-specific patient-reported outcome measure, with its psychometric properties and minimal clinically important difference validated in multicenter studies [10]. A systematic review and meta-analysis further demonstrated that patient-reported outcomes provide clinically meaningful information beyond that captured by conventional physiological indicators [11]. More importantly, recent studies have shown the independent association of QoL-B Respiratory Symptoms Scale (QoL-B-RSS) with future exacerbation risk, indicating that symptom burden is not merely subjective but also prognostically informative [12]. Therefore, outcome definitions based solely on perioperative complications may overlook patients who avoid major adverse events yet derive insufficient symptomatic benefit, whereas reliance exclusively on patient-reported outcomes may underestimate perioperative safety.

On this basis, “early unfavorable outcome” was defined as the primary endpoint by integrating Clavien-Dindo grade II or higher complications as a safety component with the QoL-B-RSS as a patient-centered benefit component, thereby constructing a composite outcome that captures both objective clinical events and subjective symptomatic recovery [10, 13, 14]. An interpretable primary model was subsequently developed using Elastic Net-penalized regression, benchmarked against a random forest (RF) model, and complemented by conventional multivariable logistic regression for enhanced interpretability. The overarching aim was to establish a clinically applicable tool for early postoperative risk stratification in patients undergoing bronchiectasis surgery.

## Methods

### Study design and participants

**Fig. 1:**
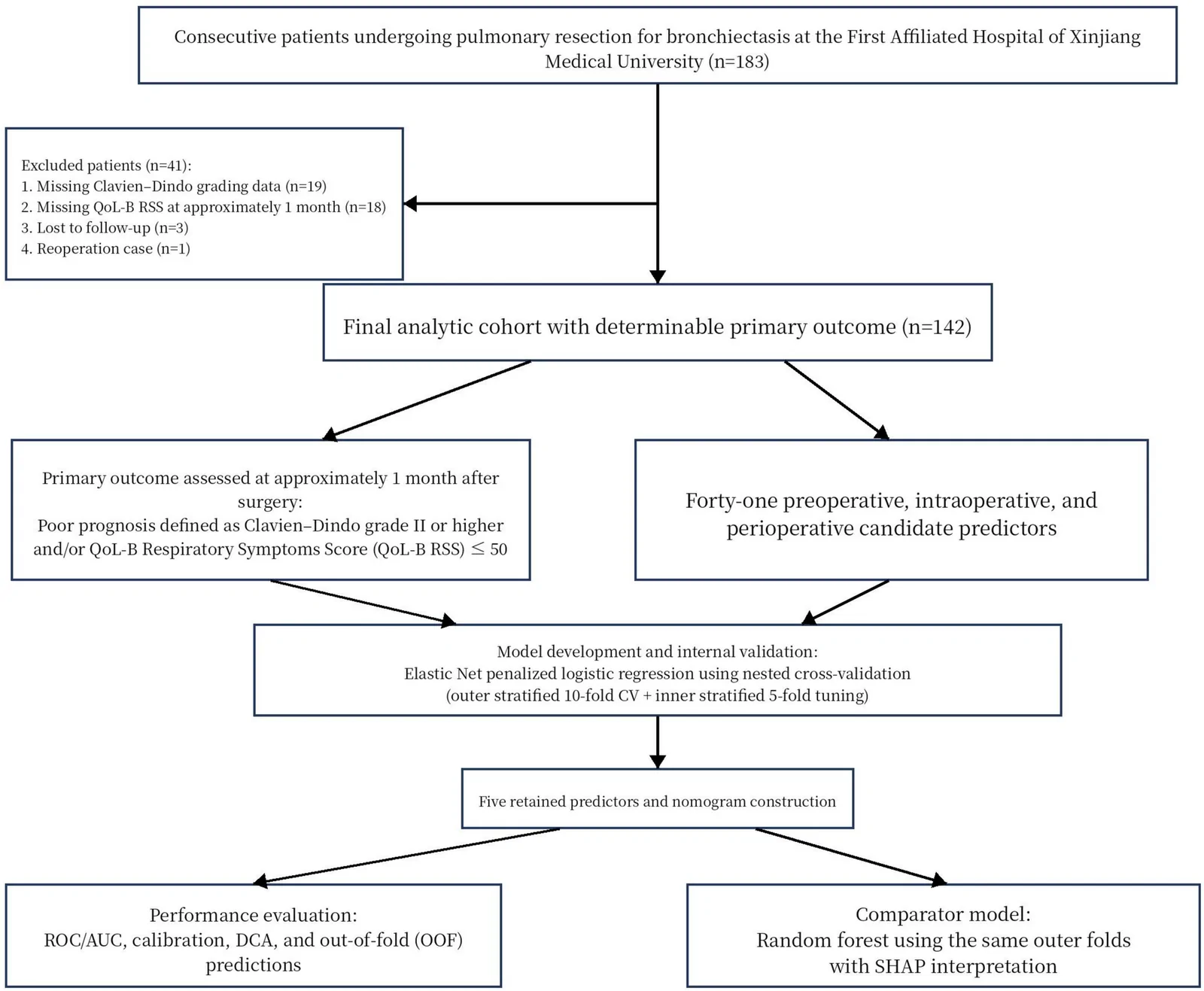
Flowchart of patient selection, outcome assessment, and model development with internal validation. Among 183 consecutive patients who underwent bronchiectasis-related lung resection, 142 were included in the analytic cohort after exclusion of 41 patients (19 without Clavien-Dindo grading, 18 without a 1-month QoL-B-RSS assessment, 3 lost to follow-up, and 1 reoperation). The primary endpoint was early unfavorable outcome at approximately 1 month postoperatively, defined as Clavien-Dindo grade II or higher complications and/or QoL-B-RSS ≤ 50. Using 40 prespecified candidate predictors, an Elastic Net model was developed and internally validated with nested cross-validation, and a random forest model was built under the same outer-fold partitions for direct comparison. QoL-B-RSS, Quality of Life Questionnaire-Bronchiectasis Respiratory Symptoms Scale

This single-center retrospective cohort study consecutively enrolled patients who underwent bronchiectasis-related lung resection at our institution between January 2020 and August 2025. Reporting adhered to the STROCSS 2021 statement, with additional details on model development and internal validation provided in accordance with the TRIPOD + AI framework [15, 16]. A total of 183 consecutive patients were initially screened. Inclusion criteria were: (1) confirmed diagnosis of bronchiectasis and treatment with lung resection; (2) availability of follow-up data sufficient for primary outcome adjudication; and (3) availability of key clinical variables. Exclusion criteria were: (1) inability to ascertain the primary outcome; (2) loss to follow-up precluding assessment of 1-month postoperative outcomes; (3) postoperative pathology inconsistent with isolated bronchiectasis; and (4) reoperation. Forty-one patients were excluded because the primary composite outcome could not be reliably adjudicated, including 19 patients with missing or incomplete Clavien-Dindo grading, 18 without 1-month QoL-B-RSS assessment, three lost to follow-up, and one who underwent reoperation before the scheduled outcome assessment. Ultimately, 142 patients with ascertainable outcomes comprised the analytic cohort. The screening process and reasons for exclusion are detailed in Fig. 1.

### Data collection

Study variables were extracted from the electronic medical record system, preoperative assessment databases, and anesthesia/operative records, consistent with the available dataset fields. Variables included: (1) demographic and baseline characteristics (e.g., sex, age, body mass index [BMI]); (2) medical history and preoperative comorbidities (e.g., tuberculosis-related history, hypertension, diabetes, and heart disease); (3) preoperative laboratory, blood gas, and biochemical parameters (e.g., hematologic indices, albumin, liver and renal function, electrolytes, glucose, pH, pCO₂, and pO₂); (4) cardiopulmonary function and perioperative risk assessments (e.g., pulmonary function indices, echocardiographic parameters, and American Society of Anesthesiologists grade); (5) disease phenotype and severity indicators (e.g., bronchiectasis phenotype and Bronchiectasis Severity Index(BSI)-related variables); and (6) surgery-related factors (e.g., operative approach, extent of resection, operative duration, intraoperative blood loss, thoracoscopic assistance, and postoperative complications). Preoperative blood samples were obtained in all patients as part of routine assessment: emergency cases were sampled on the day of surgery, whereas elective cases underwent laboratory testing on the first day after admission. After discharge, patients were followed for approximately 1 month. During this period, complications were recorded and graded according to the Clavien-Dindo classification, and the QoL-B-RSS was administered via telephone follow-up [10, 13].

### Outcome definition and composite endpoint

The primary outcome, termed “early unfavorable outcome,” was defined as a composite endpoint integrating perioperative safety and patient-perceived symptomatic benefit within the same postoperative time frame. A favorable outcome was defined as the absence of Clavien-Dindo grade II or higher complications together with a QoL-B-RSS score > 50; all other cases were classified as unfavorable outcomes. Complication detection and attribution were based on thin-slice computed tomography performed at approximately 1 month postoperatively, clinical course documentation, laboratory findings, and treatment/intervention records. Complication severity was standardized using the Clavien-Dindo classification, which is based on treatment intensity and offers strong clinical interpretability and reproducibility [13, 17]. Symptomatic benefit was assessed using the QoL-B-RSS at approximately 1 month postoperatively during outpatient visits or telephone follow-up. The QoL-B has been psychometrically validated in multiple studies as a disease-specific patient-reported outcome measure [10]. Moreover, lower QoL-B-RSS scores have been associated with an increased risk of future exacerbations [12, 18]. Therefore, QoL-B-RSS was incorporated as the patient-centered component of the composite endpoint to capture patient-reported respiratory symptom burden as part of early unfavorable outcome [10, 14]. Because no postoperative recovery-specific MCID or universally accepted QoL-B-RSS cutoff has been established for patients undergoing bronchiectasis surgery, a score of ≤ 50 was prespecified as an operational threshold for this patient-reported component. Sensitivity analyses using alternative cutoffs of ≤ 40 and ≤ 60 showed generally consistent model performance(Additional file1).

### Statistical analysis

Statistical analyses followed current methodological recommendations for clinical prediction model development [16, 19–23]. Continuous variables with approximate normal distributions are presented as mean ± standard deviation and were compared using Student’s t-test. Non-normally distributed continuous variables are reported as median (Q1, Q3) and were compared using the Mann-Whitney U test. Categorical variables are summarized as counts (percentages) and were compared using the χ² test or Fisher’s exact test, as appropriate. A two-sided P value < 0.05 was considered statistically significant. Baseline comparisons were conducted solely for descriptive purposes and were not used for variable selection or model construction. To minimize bias associated with data-driven selection, all 40 candidate predictors were prespecified based on clinical interpretability, prior literature, and availability before surgery, rather than being screened using univariable P values [19–22]. All analyses were performed via IBM SPSS Statistics 26.0 and R 4.3.2.

### Model development and internal validation

A clinical prediction model for early unfavorable outcomes was developed using the prespecified predictors. To prevent information leakage and enhance reproducibility, all preprocessing and modeling steps were implemented within a unified pipeline, with parameters estimated exclusively from training data before application to validation data. Continuous variables were imputed and standardized using training-derived parameters, while categorical variables underwent missing-data handling and one-hot encoding based on training-set rules.

Elastic Net-penalized logistic regression was selected as the primary modeling approach to achieve coefficient shrinkage while maintaining interpretability. Following internal validation, a nomogram was constructed from the final model [16, 19, 20, 24]. To obtain a robust estimate of internal validation performance and reduce optimism from model selection, nested stratified cross-validation was used. A stratified 10-fold outer loop was applied to generate out-of-fold predicted probabilities, while a stratified 5-fold inner loop within each outer training set was used for grid-search tuning of the regularization parameters. To minimize information leakage, all preprocessing steps were fitted within the training folds and subsequently applied to the corresponding validation folds. The mean area under the curve (AUC) was used as the primary criterion for hyperparameter selection, with the Brier score considered as a secondary criterion when model performance was comparable. Predicted probabilities from outer-loop test folds were aggregated to generate out-of-fold (OOF) predicted probabilities, which were used to evaluate discrimination, overall prediction error, calibration, and clinical utility using decision curve analysis (DCA). After the final Elastic Net model specification had been determined, bootstrap optimism correction with 1,000 resamples was additionally performed to estimate optimism-corrected AUC, Brier score, and calibration slope. For nomogram construction, the primary model was refitted on the full dataset using the optimal penalization parameters, and a nomogram was derived from the final coefficients [19, 20, 24, 25].

To assess potential gains from modeling nonlinear relationships and interactions, a random forest (RF) model was developed as a comparator. Both models used identical outer-loop partitions and evaluation procedures to ensure a fair comparison. Hyperparameters for the RF were tuned within training folds, and probability calibration was applied when necessary before evaluation. Model performance was assessed using the same metrics (AUC, Brier score, calibration, and DCA), and differences in performance were quantified. For interpretability, the nomogram served as the primary explanatory output for the Elastic Net model, while Shapley Additive exPlanations (SHAP) analysis was used to complement the interpretation of the RF model (Supplementary Figure S1) [19, 20, 25–28].

### Supplementary sensitivity analysis

Following primary model development, correlations among retained predictors were examined. Where clinically related predictors with relatively high correlations were identified, supplementary sensitivity analyses were conducted without altering the dataset, outcome definition, cross-validation scheme, or evaluation framework. Specifically, alternative models were constructed by sequentially removing one variable at a time from the correlated set. Model performance (AUC and Brier score; Table S1) was compared with that of the primary model to evaluate robustness and the relative contribution of each predictor [28].

## Results

### Patient characteristics and composition of the composite endpoint

A total of 142 patients who underwent bronchiectasis-related lung resection were included, of whom 60 had favorable outcomes, and 82 had unfavorable outcomes, corresponding to an early unfavorable outcome rate of 57.7%. Within the composite endpoint, 77 patients experienced Clavien-Dindo grade II or higher complications, and 31 had a QoL-B-RSS score ≤ 50. Among these, 26 patients met both criteria, 51 had complications only, and five had impaired QoL-B-RSS only. These findings indicate that early postoperative recovery is multidimensional, with only partial overlap between perioperative complications and suboptimal symptomatic recovery. Reliance on a single complication-based or patient-reported endpoint would therefore fail to identify a subset of high-risk patients. The composite endpoint was therefore adopted as the primary outcome to more comprehensively characterize overall early postoperative recovery after bronchiectasis surgery.

Baseline characteristics were descriptively compared between outcome groups (Table 1). Overall, patients with unfavorable outcomes exhibited poorer preoperative ventilatory and pulmonary reserve, greater disease burden, and more complex perioperative features. Specifically, the unfavorable group had higher preoperative pCO₂ levels [40.80 (38.00, 46.75) vs. 37.75 (36.00, 39.25), *P* < 0.001], worse pulmonary function, including lower forced expiratory volume in 1 s as a percentage of predicted (FEV1%pred) [62.00 (56.00, 84.07) vs. 89.88 (80.45, 97.25), *P* < 0.001] and maximal voluntary ventilation as a percentage of predicted (MVV%pred) [54.19 (46.45, 63.26) vs. 73.59 (63.87, 82.34), *P* < 0.001], and a higher total BSI score [6 (4, 7) vs. 3.00 (2.00, 4.25), *P* < 0.001]. Perioperatively, local pleural changes were more frequent, operative duration was longer, and thoracoscopic assistance was less commonly used in the unfavorable group. Collectively, these patterns suggest that early unfavorable outcomes may reflect the combined influence of baseline disease severity, cardiopulmonary reserve, and operative complexity, although independent effects require further model-based evaluation.

**Table 1:** Baseline characteristics of patients with favorable and unfavorable outcomes after bronchiectasis surgery

Variable	Prognosis good (No)	Poor prognosis (Yes)	*P* value
Gender			
Female	38(63.3%)	47(57.3%)	0.583
Male	22(36.7%)	35(42.7%)	
Age	46(30, 56)	52.50(38.25, 58.75)	0.101
BMI Index	23.74 ± 4.09	23.31 ± 3.60	0.519
Body Surface Area (㎡)	1.70(1.60, 1.86)	1.72(1.56, 1.86)	0.596
Disease duration (years)	1.00(0.23, 4.25)	2.00(0.35, 7.75)	0.145
Heart Disease History	3(5.0%)	6(7.3%)	0.734
Diabetes History	2(3.3%)	7(8.5%)	0.302
Hypertension History	4(6.7%)	9(11.0%)	0.559
Prior Thoracic Surgery	1(1.7%)	10(12.2%)	0.025
Preoperative Neutrophil Percentage	54.95(51.00, 63.33)	57.70(51.00, 64.90)	0.382
Preoperative Hemoglobin (g/L)	130.00(120.75, 139.00)	127.00(115.25, 141.75)	0.330
Preoperative Blood Gas pH	7.39(7.37, 7.42)	7.38(7.36, 7.41)	0.064
Preoperative pCO2 (mmHg)	37.75(36.00, 39.25)	40.80(38.00, 46.75)	< 0.001
Preoperative pO2 (mmHg)	84.15(78.75, 93.00)	81.85(75.00, 92.00)	0.139
Preoperative Creatinine (µmol/L)	58.00(50.00, 69.25)	58.50(48.10, 68.96)	0.934
Preoperative Potassium (mmol/L)	3.55(3.35, 3.78)	3.62(3.35, 3.81)	0.673
Preoperative serum sodium (mmol/L)	140.31(138.86, 142.20)	140.34(138.16, 142.00)	0.510
Preoperative serum albumin (g/L)	40.00(37.11, 42.50)	39.51(37.60, 42.36)	0.797
Preoperative ALT (U/L)	21(15, 29)	20(16, 26)	0.601
Preoperative AST (U/L)	25.00(21.95, 30.00)	23.00(20.00, 26.50)	0.068
Preoperative Glucose (mmol/L)	5.32(4.51, 6.37)	5.26(4.86, 6.10)	0.658
Preoperative Symptoms			
Hemoptysis	6(10.0%)	11(13.4%)	0.220
Hemoptysis and Infection	4(6.7%)	9(11.0%)	
Infection	34(56.7%)	32(39.0%)	
Other Chest Diseases	13(21.7%)	16(19.5%)	0.917
Complete resection of diseased lesions	37(61.7%)	44(53.7%)	0.435
Surgery Duration (h)	2.50(2.00, 3.00)	3.00(2.50, 4.00)	0.011
Number of involved lobes	2(1, 2)	2(1, 3)	0.399
VATS assistance	59(98.3%)	68(82.9%)	0.013
ASA Classification			
Class 2	34(56.7%)	42(51.2%)	0.637
Class 3	26(43.3%)	40(48.8%)	
Duration of Intraoperative Endotracheal Intubation Assisted Ventilation (h)	3.50(2.50, 4.00)	3.75(3.00, 4.12)	0.305
VTE High Risk (≥ 5)	4(6.7%)	5(6.1%)	1.000
Type of Bronchial Dilatation Structure			
Cystic	38(63.3%)	43(52.4%)	0.053
Cylindrical	7(11.7%)	4(4.9%)	
Cystic and Cylindrical Mixed	15(25.0%)	35(42.7%)	
Intraoperative Pleural Local Changes	25(41.7%)	59(72.0%)	< 0.001
Left Ventricular Ejection Fraction	63(62, 64)	63.00(61.90, 65.00)	0.443
Pulmonary Artery Diameter (mm)	23(22, 24)	22(21, 24)	0.568
Abnormal Electrocardiogram Results	11(18.3%)	14(17.1%)	1.000
FEV1%pred	89.88(80.45, 97.25)	62.00(56.00, 84.07)	< 0.001
FEV1/FVC %pred	94.88(89.29, 99.10)	86.09(79.00, 92.32)	< 0.001
MVV%pred	73.59(63.87, 82.34)	54.19(46.45, 63.26)	< 0.001
BSI Grade			
Moderate	13(21.7%)	38(46.3%)	< 0.001
Mild	45(75.0%)	32(39.0%)	
Severe	2(3.3%)	12(14.6%)	
BSI Total Score	3.00(2.00, 4.25)	6(4, 7)	< 0.001

Data are presented as n (%), mean ± SD, or median (Q1, Q3), as appropriate. Group comparisons were performed using the χ² test or Fisher’s exact test for categorical variables, Student’s t test for normally distributed continuous variables, and the Mann-Whitney U test for non-normally distributed continuous variables. Bold values indicate statistically significant differences (*P* < 0.05)

*ALT *Alanine aminotransferase, *ASA* American Society of Anesthesiologists, *AST* Aspartate aminotransferase, *BMI* Body mass index, *BSI* Bronchiectasis Severity Index, *FEV1%pred* Forced expiratory volume in 1 s as percentage of predicted, *FEV1/FVC %pred* Ratio of forced expiratory volume in 1 s to forced vital capacity as percentage of predicted, *MVV%pred* Maximal voluntary ventilation as percentage of predicted, *pCO2* Partial pressure of carbon dioxide, *pO2* Partial pressure of oxygen, *VATS* Video-assisted thoracoscopic surgery, *VTE* Venous thromboembolism

### Model development based on candidate predictors

Elastic Net-penalized logistic regression was used as the primary modeling approach, enabling variable selection and stable coefficient estimation through shrinkage. The final model retained five predictors: preoperative pCO₂, left ventricular ejection fraction (LVEF), FEV1%pred, MVV%pred, and total BSI score. These variables represent three principal domains of risk: disease burden, ventilatory/pulmonary reserve, and overall cardiopulmonary reserve. To facilitate clinical interpretability, a conventional multivariable logistic regression model was additionally fitted using the retained predictors. Preoperative pCO₂ was independently associated with increased risk of unfavorable outcome (OR = 2.84, 95% CI 1.46–5.56, *P* = 0.002), FEV1%pred was protective (OR = 0.26, 95% CI 0.10–0.70, *P* = 0.008), and total BSI score was positively associated with risk (OR = 1.77, 95% CI 1.06–2.96, *P* = 0.030). MVV%pred and LVEF showed effect directions consistent with the primary model (Figure S2).

### Construction and evaluation of the nomogram

**Fig. 2:**
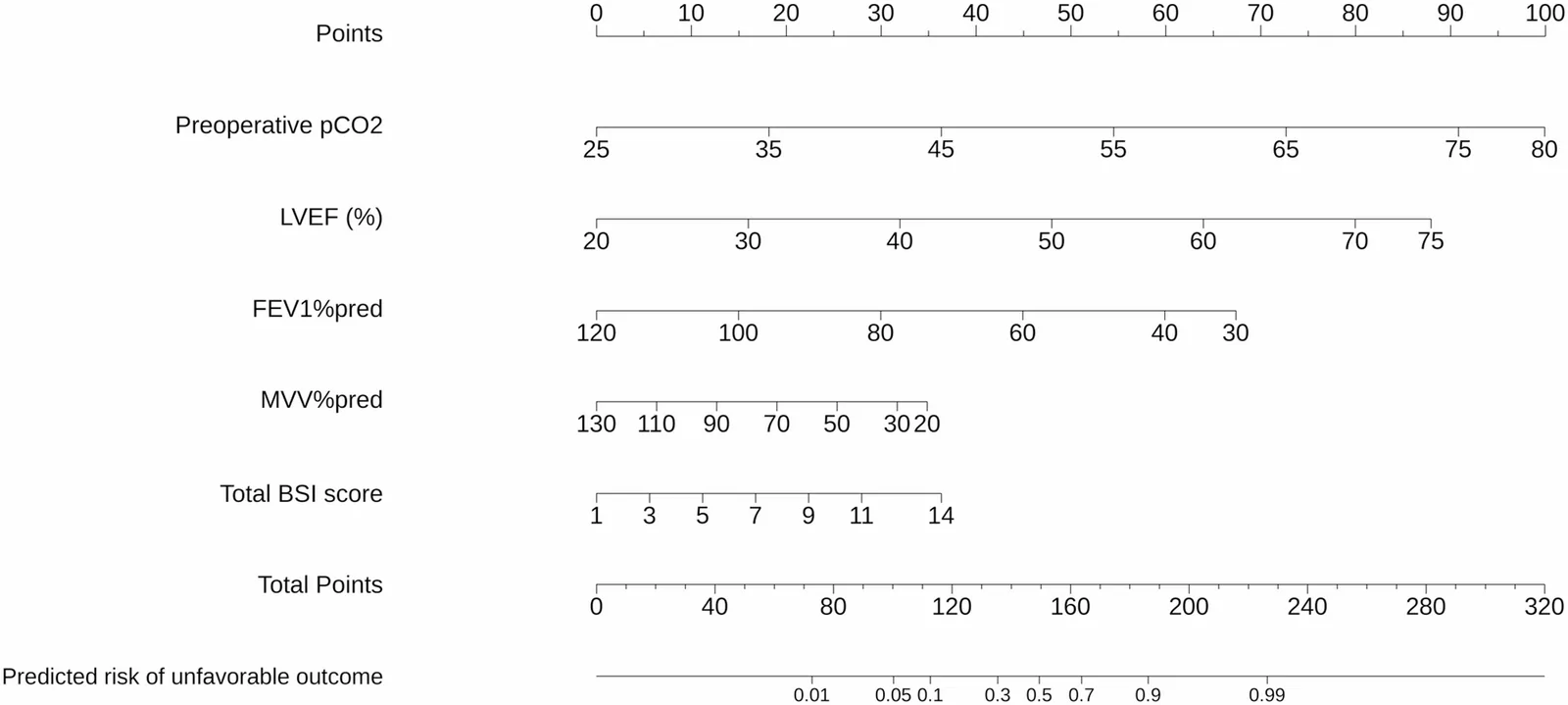
Nomogram for predicting early unfavorable outcome after bronchiectasis surgery. This nomogram was constructed from the final Elastic Net penalized logistic regression model to provide individualized prediction of early unfavorable outcome after bronchiectasis surgery. The nomogram includes five retained predictors: preoperative pCO2, left ventricular ejection fraction (LVEF), FEV1%pred, MVV%pred, and total Bronchiectasis Severity Index (BSI) score. For each predictor, a point value is assigned according to the patient’s value on the corresponding scale. The points for all predictors are then summed and projected onto the bottom risk scale to estimate the individualized probability of early unfavorable outcome

**Fig. 3:**
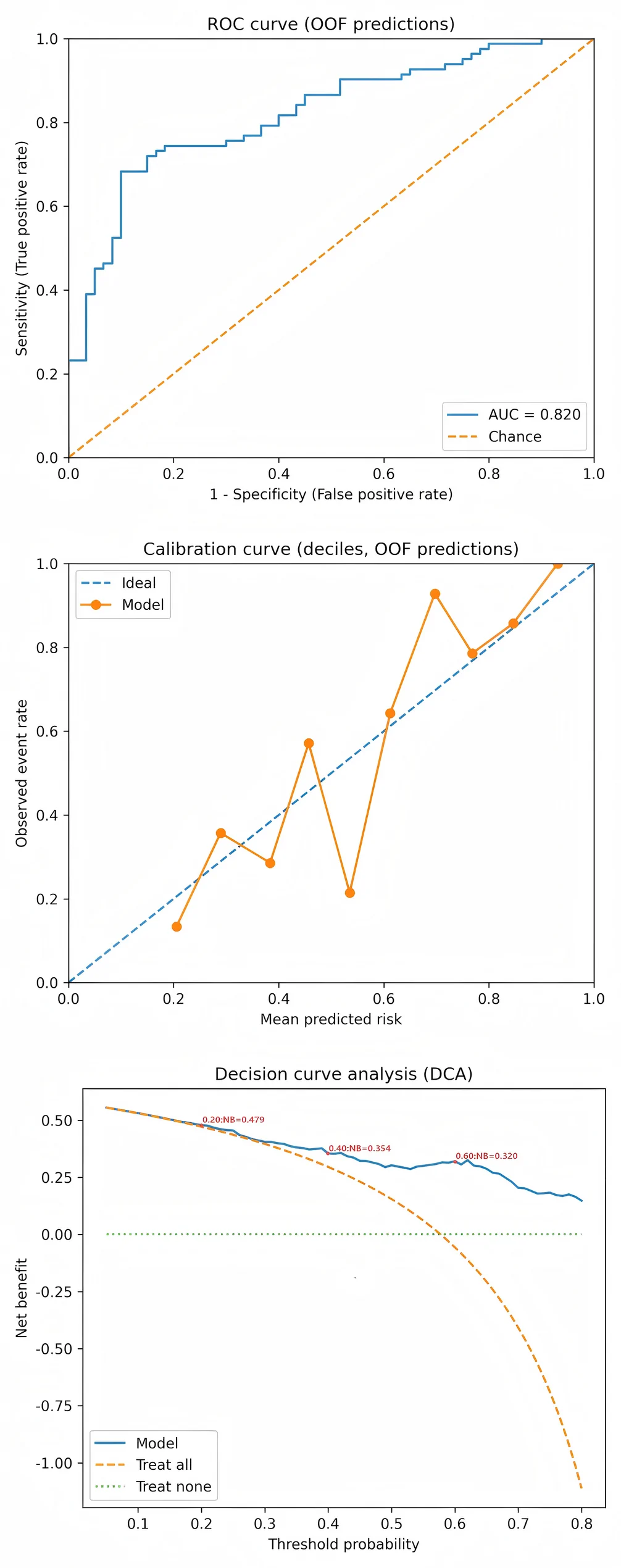
Performance of the final Elastic Net model in internal validation. **A** Receiver operating characteristic (ROC) curve based on out-of-fold (OOF) predictions, showing an area under the curve (AUC) of 0.820. The OOF Brier score was 0.173. **B** Calibration curve based on OOF predictions, with observed event rates plotted against predicted probabilities by deciles of risk. Quantitative calibration assessment showed a calibration intercept of -0.018, calibration slope of 1.279, calibration-in-the-large of 0.026, and observed-to-expected ratio of 1.009. **C** Decision curve analysis (DCA) showing the net benefit of the Elastic Net model relative to the treat-all and treat-none strategies across the evaluated threshold probability range of 0.05 to 0.80. Numerical annotations are shown at selected threshold probabilities to illustrate the magnitude of net benefit: the model net benefit was 0.479 at a threshold probability of 0.20, 0.354 at 0.40, and 0.320 at 0.60. These threshold probabilities were used for decision-analytic interpretation and not as mandatory clinical intervention cutoffs

A nomogram for predicting early unfavorable outcomes after bronchiectasis surgery was constructed based on the final Elastic Net model (Fig. 2), incorporating preoperative pCO₂, LVEF, FEV1%pred, MVV%pred, and total BSI score (Table 2). Each predictor corresponds to a point value, and the total score can be mapped to an individualized predicted probability of an unfavorable outcome. In general, higher pCO₂ and BSI scores increase predicted risk, whereas better pulmonary function (e.g., higher FEV1%pred) is associated with lower risk. Within the nested cross-validation framework, the Elastic Net model achieved an OOF AUC of 0.820 and an OOF Brier score of 0.173, indicating good discriminative ability and acceptable overall prediction error. Quantitative calibration assessment based on OOF predictions showed a calibration intercept of -0.018, calibration slope of 1.279, calibration-in-the-large of 0.026, and observed-to-expected ratio of 1.009. Bootstrap optimism correction yielded an optimism-corrected AUC of 0.824, Brier score of 0.174, and calibration slope of 1.123(Additional file2). DCA indicated potential net clinical benefit across the evaluated threshold probability range of 0.05 to 0.80 (Fig. 3).

**Table 2:** Coefficients of the final Elastic Net model

Variable	Coefficient β (per 1 SD)	OR (per 1 SD)
(Intercept)	0.471579045	1.602522652
Preoperative pCO2 (mmHg)	0.540494502	1.71685564
Left Ventricular Ejection Fraction	0.053596191	1.055058474
FEV1%pred	-0.912689209	0.401443206
MVV%pred	-0.061027873	0.940797016
BSI Total Score	0.251482904	1.285930916

Except for the intercept, all continuous predictors were standardized by the sample standard deviation (SD) before model fitting. Accordingly, β coefficients and ORs represent the change in log-odds and odds of early unfavorable outcome associated with a 1-SD increase in the corresponding predictor. This standardization places continuous predictors on a comparable scale across different measurement units

*OR* Odds ratio, *SD* Standard deviation

### Comparator model and supplementary sensitivity analysis

**Fig. 4:**
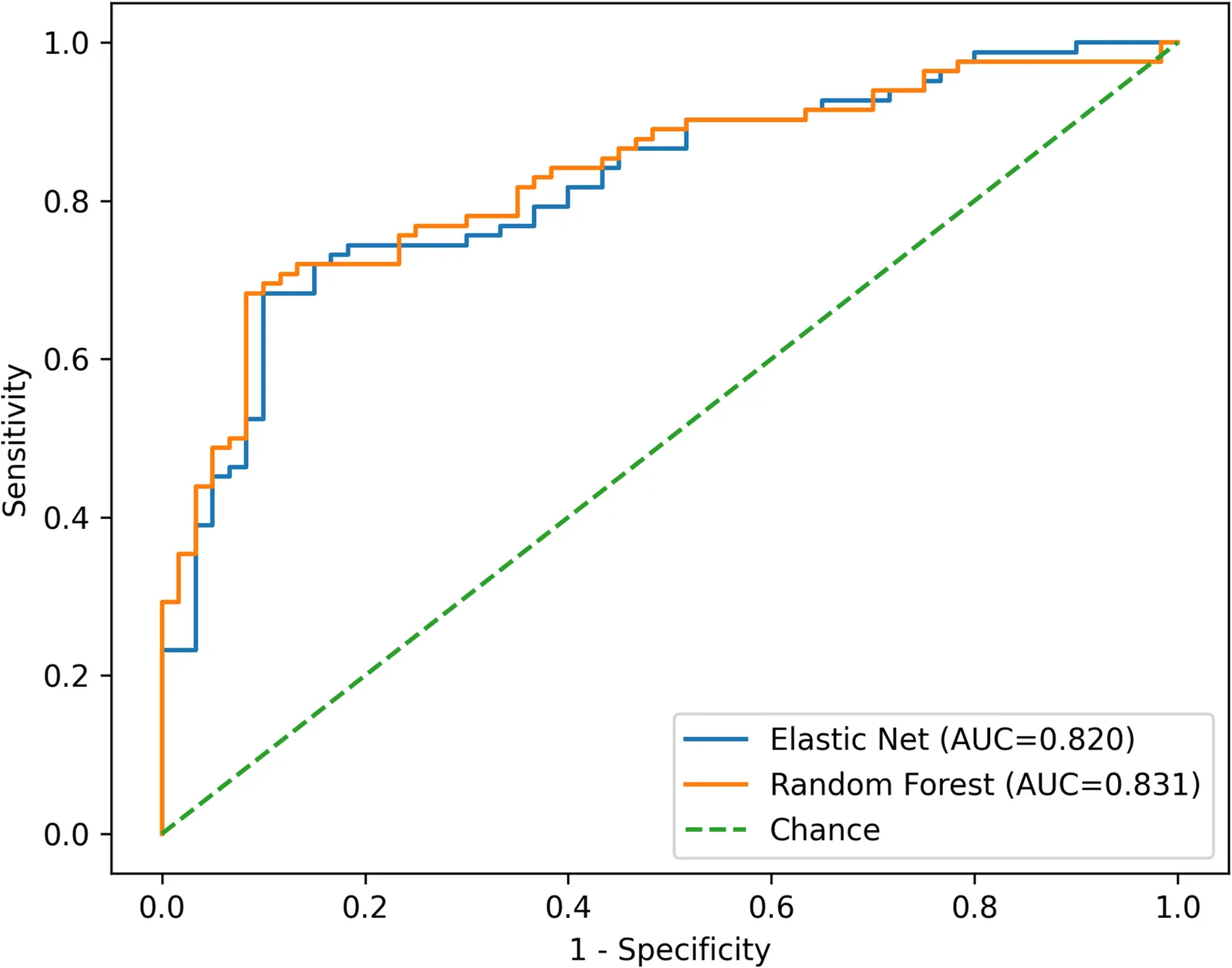
ROC comparison of the Elastic Net and random forest models in internal validation. ROC curves based on OOF predictions showed similar discrimination for the Elastic Net model (AUC = 0.820) and the random forest model (AUC = 0.831). Both models were evaluated within the same nested cross-validation framework and identical outer-fold partitions to enable a fair comparison

Within the same OOF framework, the RF model achieved an AUC of 0.831, slightly higher than that of the primary model, but with a Brier score of 0.179, which did not outperform the primary model (Table 3; Fig. 4). These findings suggest that, given the current sample size and relatively low-dimensional predictor set, the more complex nonlinear model did not confer a meaningful performance advantage. The modest improvement in discrimination was insufficient to offset reduced interpretability and greater implementation complexity, supporting the use of the penalized regression-based nomogram as the primary clinical tool.

**Table 3:** Comparison of model performance based on out-of-fold predictions

Model	AUC (OOF)	Brier (OOF)	Specificity	PPV
Elastic Net Logistic	0.82	0.173	0.90	0.90
Random Forest	0.83	0.179	0.92	0.92

Data are presented as numerical values. A higher AUC indicates better discrimination, whereas a lower Brier score indicates better overall prediction error. *AUC* Area under the receiver operating characteristic curve, *OOF* Out-of-fold, *PPV* Positive predictive value

Sensitivity analyses were conducted by sequentially removing FEV1%pred and MVV%pred and comparing the resulting models with the primary model. Exclusion of either variable resulted in only minimal changes in model performance, further supporting the robustness and stability of the primary model despite the inclusion of these correlated predictors.

## Discussion

This study developed and internally validated an interpretable prognostic model for overall early recovery approximately 1 month after lung resection for bronchiectasis using a composite endpoint. Three key findings warrant emphasis. First, early unfavorable outcomes were common, occurring in 57.7% of patients, underscoring substantial heterogeneity in short-term postoperative recovery even among surgically selected individuals. Second, Clavien-Dindo grade II or higher complications and QoL-B-RSS ≤ 50 exhibited only partial overlap, indicating that a single endpoint is insufficient to capture the multidimensional nature of postoperative recovery. Third, when discrimination, probability error, and clinical interpretability were jointly considered, the Elastic Net-derived nomogram appeared more suitable than the RF model for clinical application.

Within the composite endpoint, 77 patients experienced Clavien-Dindo grade II or higher complications, and 31 had QoL-B-RSS ≤ 50; only 26 met both criteria, whereas 51 had complications alone and five had isolated poor symptomatic recovery. This distribution suggests that complication-based endpoints may overlook patients who recover uneventfully yet derive insufficient symptomatic benefit, while patient-reported outcomes alone may underestimate objective safety concerns. Therefore, within the management frameworks emphasized by the European Respiratory Society and British Thoracic Society, namely, reducing symptom burden, preventing exacerbations, and improving quality of life [1, 2], the integration of Clavien-Dindo grading and QoL-B-RSS provides a more balanced representation of both surgical safety and patient-centered benefit after bronchiectasis surgery [10–14].

This composite approach is also aligned with recent high-quality reviews advocating for integrated management strategies that address not only acute clinical events but also symptom burden and quality-of-life outcomes [4, 5, 11]. The QoL-B is a bronchiectasis-specific patient-reported outcome instrument in which domain scores are linearly transformed to a 0-100 scale, with higher scores indicating lower symptom burden or better functional status; its psychometric properties have been validated in multicenter studies. Prior research further indicates that QoL-B-RSS is independently associated with future exacerbation risk and that values ≤ 50 may reflect a higher symptom burden [10, 12, 18].

It should be noted that the QoL-B-RSS cutoff used in this study was a prespecified operational threshold for the patient-reported component of the composite endpoint, rather than a formally validated postoperative recovery threshold. Although sensitivity analyses using QoL-B-RSS cutoffs of ≤ 40, ≤50, and ≤ 60 showed generally consistent model performance, dichotomization of a continuous patient-reported outcome may lead to loss of information. Moreover, a fixed postoperative cutoff may not perform uniformly across patients with different baseline symptom burdens or disease severity. Future prospective studies should collect longitudinal preoperative and postoperative QoL-B-RSS data and evaluate continuous, ordinal, or change-based definitions of symptomatic recovery.

Previous studies have demonstrated that appropriately selected patients with localized bronchiectasis may experience symptom relief and improved quality of life after lung resection, although perioperative complications and recovery trajectories vary considerably [6, 7, 29]. In contrast to prior work focusing primarily on individual complications (e.g., postoperative pneumonia) or isolated risk domains such as nutritional or inflammatory status [9, 30, 31], the present study approached postoperative prognosis from the perspective of “early unfavorable outcome.”

The findings indicate that elevated preoperative pCO₂, together with reduced FEV1%pred and MVV%pred, reflects impaired ventilatory and pulmonary reserve. This is pathophysiologically plausible: elevated pCO₂ often indicates reduced alveolar ventilation efficiency or impaired ventilation-perfusion matching, whereas lower FEV1%pred and MVV%pred suggest limited ventilatory reserve and diminished airway clearance capacity [32–36]. In patients with bronchiectasis, such impairments are more likely to translate into increased complications or delayed symptomatic recovery under the physiological stress of anesthesia, lung resection, and postoperative secretion retention [29, 33, 37].

Beyond respiratory reserve, the inclusion of LVEF and BSI in the final model suggests that overall compensatory capacity and disease burden also influence early postoperative recovery. The BSI is an internationally validated severity index that robustly predicts mortality, hospitalization, and exacerbation risk [38, 39]. Although its role in postoperative stratification has shown variability across surgical studies [40], the present findings, indicating poorer early recovery among patients with higher BSI, support its relevance in the perioperative context.

With respect to LVEF, prior studies in noncardiac and thoracic surgery have linked impaired cardiac function to increased perioperative risk [41–44]. However, given that the statistical evidence for LVEF was less robust than that for pCO₂, FEV1%pred, and total BSI score in the conventional multivariable model, it is more appropriate to interpret LVEF as a marker of global cardiopulmonary reserve rather than a definitive independent predictor.

Overall, these findings support a more integrated framework for surgical risk assessment in bronchiectasis that extends beyond technical resectability to encompass disease burden, ventilatory reserve, and global cardiopulmonary function.

From a methodological perspective, Elastic Net was selected as the primary modeling approach and was compared with an RF model using the same nested cross-validation and OOF evaluation framework. The Elastic Net model achieved an OOF AUC of 0.820 and an OOF Brier score of 0.173, whereas the RF model achieved a slightly higher OOF AUC of 0.831 but a higher Brier score of 0.179. These findings suggest that, in the current dataset with a modest sample size and a relatively limited predictor set, the more complex nonlinear RF model did not provide a clear overall performance advantage. The small numerical gain in discrimination was therefore not sufficient to outweigh the reduced interpretability and greater implementation complexity of RF. In contrast, the Elastic Net model offered a more transparent and clinically usable framework for nomogram construction.

Although nested cross-validation, OOF evaluation, and bootstrap optimism correction supported the internal robustness of the primary model, external validation in independent cohorts is still needed to further confirm its reliability and generalizability. [16, 19, 20, 25, 27, 28].

Therefore, the penalized regression-based nomogram may provide a transparent and interpretable framework for perioperative risk stratification. Rather than defining a fixed intervention threshold, the predicted risk should be interpreted as a tool to inform risk-stratified perioperative management after prospective external validation. Patients estimated to be at higher risk, particularly those with elevated pCO₂, impaired pulmonary reserve, reduced cardiopulmonary reserve, or higher BSI scores, may be considered for closer postoperative monitoring, optimized airway clearance strategies, respiratory prehabilitation, perioperative cardiopulmonary optimization, and more structured early follow-up. Decisions regarding higher-level monitored care, including high-dependency or intensive care observation, should remain individualized and based on the overall clinical context rather than on a single model-derived cutoff.

This interpretation is consistent with contemporary bronchiectasis management principles emphasizing airway clearance, mucoactive therapy, and pulmonary rehabilitation [45].

There are several limitations. First, this was a single-center retrospective study, and 41 of the 183 initially screened patients were excluded due to incomplete outcome data, unavailable follow-up, or reoperation before the scheduled outcome assessment. Because the analytic cohort was defined after confirming outcome availability, complete baseline and perioperative variables were not available for excluded patients, precluding a formal included-versus-excluded baseline comparison. Therefore, complete-case analysis may have introduced selection bias, particularly if missing outcome information was related to follow-up adherence, postoperative recovery, symptom burden, or complication severity [19–22].

Second, the sample size was modest. Although nested cross-validation and OOF prediction reduce optimism, the findings are based on internal validation and require confirmation through external validation in larger, multicenter cohorts.

Third, the use of QoL-B-RSS > 50 as part of the composite endpoint represents a pragmatic operational threshold for early postoperative recovery and should not be interpreted as a universal cutoff or a surrogate for long-term outcomes [10, 12, 18].

Finally, the one-month postoperative time frame primarily reflects early recovery and cannot substitute for the evaluation of long-term outcomes, including recurrent infections, sustained symptom control, postoperative pain, anxiety and quality-of-life improvement.

Nevertheless, the present findings indicate that an interpretable model based on preoperatively available variables can effectively identify patients at high risk of early unfavorable recovery following bronchiectasis surgery, thereby providing quantitative support for perioperative management and individualized follow-up strategies.

## Conclusions

An interpretable Elastic Net-based prognostic model was developed and internally validated to predict early unfavorable outcomes after bronchiectasis surgery. Within a rigorous OOF evaluation framework, the model demonstrated good discrimination, acceptable calibration error, and potential clinical utility. The derived nomogram may facilitate individualized risk assessment and provide quantitative guidance for perioperative risk stratification, management optimization, and early postoperative follow-up. Further validation in larger samples and external cohorts is warranted to confirm its robustness and generalizability.

## Supplementary Information

Supplementary Material 1. Additional file 1. Composite Endpoint and QoL-B-RSS Sensitivity Analysis.Additional file 2: Internal Validation, Model Development, DCA, and RF Comparator. Additional file 3: FEV1%pred and MVV%pred Collinearity Analysis. Additional file 4: Exclusion Reasons and Candidate Predictor Missingness. Supplementary Figure S1. SHAP summary plot of the top 10 features in the random forest comparator model. This figure presents the 10 most influential features in the random forest comparator model, ranked by mean absolute SHAP value. Each dot represents one patient. The horizontal position indicates the direction and magnitude of that feature's contribution to the predicted risk of early unfavorable outcome; positive SHAP values indicate a higher predicted risk and negative SHAP values indicate a lower predicted risk. Dot color represents the original feature value, with red denoting higher values and blue denoting lower values. Features are ordered from top to bottom according to mean absolute SHAP value, with higher-ranked features contributing more to the overall model prediction. SHAP, Shapley Additive exPlanations. Supplementary Figure S2. Forest plot of odds ratios from conventional multivariable logistic regression including the predictors retained in the Elastic Net model. Dots represent odds ratios (ORs), and horizontal bars represent 95% confidence intervals (95% CIs) from a conventional multivariable logistic regression including the five predictors retained in the Elastic Net model: preoperative pCO2, left ventricular ejection fraction (LVEF), forced expiratory volume in 1 second as percentage of predicted (FEV1%pred), maximal voluntary ventilation as percentage of predicted (MVV%pred), and total Bronchiectasis Severity Index (BSI) score. All continuous predictors were entered per 1-standard deviation increase. The vertical dashed line indicates the null value of OR = 1.0. OR > 1 indicates an increased risk of early unfavorable outcome, whereas OR < 1 indicates a decreased risk. In this supplementary analysis, preoperative pCO2 and total BSI score were associated with higher risk, FEV1%pred showed a protective association, and LVEF and MVV%pred showed directions consistent with the primary model. Supplementary Table S1. Sensitivity analyses showing model performance after sequential removal of correlated predictors. Sensitivity analysis for the inclusion of FEV1%pred and MVV%pred in the primary model. The primary model was the final Elastic Net model. Alternative models were reconstructed after excluding FEV1%pred or MVV%pred separately, with all other analytic settings unchanged. Excluding FEV1%pred worsened model performance (AUC 0.821 to 0.794; Brier score 0.171 to 0.183), whereas excluding MVV%pred produced minimal change (AUC 0.822; Brier score 0.170). This suggests that FEV1%pred carried more key predictive information, while MVV%pred added relatively limited incremental value.

## Data Availability

The datasets used and/or analyzed during the current study are not publicly available due to patient privacy and institutional data-sharing restrictions but are available from the corresponding author on reasonable request.

## Abbreviations

ASA: American Society of Anesthesiologists
AUC: Area under the curve
BMI: Body mass index
BSI: Bronchiectasis Severity Index
DCA: Decision curve analysis
FEV1%pred: Forced expiratory volume in 1 second as a percentage of predicted
LVEF: Left ventricular ejection fraction
MVV%pred: Maximal voluntary ventilation as a percentage of predicted
OOF: Out-of-fold
QoL-B: Quality of Life Questionnaire-Bronchiectasis
QoL-B-RSS: Quality of Life Questionnaire-Bronchiectasis Respiratory Symptoms Scale
RF: Random forest
SHAP: Shapley Additive exPlanations
STROCSS: Strengthening the Reporting of Cohort, Cross-sectional and Case-control Studies in Surgery
TRIPOD+AI: Transparent Reporting of a Multivariable Prediction Model for Individual Prognosis or Diagnosis plus Artificial Intelligence
