# Supplementary material for: An interpretable prognostic model for early unfavorable outcomes after bronchiectasis surgery
Source: BMC Pulm Med. 2026 Jul 16;26:426. doi: 10.1186/s12890-026-04508-4 (PMC13613597; doi:10.1186/s12890-026-04508-4)
Supplement: Supplementary file 1 [file 12890_2026_4508_MOESM1_ESM.zip › Additional file 1.docx]

**Additional file 1: Composite Endpoint and QoL-B-RSS Sensitivity Analysis**

This additional file summarizes the composition of the primary composite endpoint and the sensitivity analyses using alternative QoL-B-RSS thresholds.

**Table S1. Composition of the primary composite endpoint**

| **Endpoint component** | **n** |
| --- | --- |
| Clavien-Dindo grade II or higher | 77 |
| QoL-B-RSS ≤50 | 31 |
| Both components present | 26 |
| Clavien-Dindo only | 51 |
| QoL-B-RSS only | 5 |
| Total early unfavorable outcome | 82 |
| Total analytic cohort | 142 |

*Note: The primary definition of early unfavorable outcome was Clavien-Dindo grade II or higher and/or QoL-B-RSS ≤50 approximately 1 month after surgery.*

**Table S2. Sensitivity analysis using alternative QoL-B-RSS thresholds**

| **QoL-B-RSS threshold** | **Unfavorable outcome n** | **Unfavorable outcome %** | **CD ≥II n** | **QoL-B-RSS threshold-positive n** | **Complication-only n** | **QoL-only n** | **Both n** | **Favorable n** | **AUC** | **Brier** |
| --- | --- | --- | --- | --- | --- | --- | --- | --- | --- | --- |
| ≤40 | 79 | 55.6 | 77 | 8 | 71 | 2 | 6 | 63 | 0.830 | 0.167 |
| ≤50 (primary) | 82 | 57.7 | 77 | 31 | 51 | 5 | 26 | 60 | 0.820 | 0.171 |
| ≤60 | 82 | 57.7 | 77 | 32 | 50 | 5 | 27 | 60 | 0.820 | 0.171 |

*Note: AUC and Brier score were based on out-of-fold predictions under each endpoint definition. The threshold of QoL-B-RSS ≤50 was the primary definition.*
