# Supplementary material for: An interpretable prognostic model for early unfavorable outcomes after bronchiectasis surgery
Source: BMC Pulm Med. 2026 Jul 16;26:426. doi: 10.1186/s12890-026-04508-4 (PMC13613597; doi:10.1186/s12890-026-04508-4)
Supplement: Supplementary file 1 [file 12890_2026_4508_MOESM1_ESM.zip › Additional file 2.docx]

**Additional file 2: Internal Validation, Model Development, DCA, and RF Comparator**

This additional file summarizes model development, internal validation, calibration, decision-curve analysis, threshold interpretation, Random Forest comparison, and per-fold performance.

**Table S1. Model development and preprocessing specifications**

| **Item** | **Specification** |
| --- | --- |
| Software environment | Descriptive statistics and conventional statistical analyses were performed using IBM SPSS Statistics version 26.0. Model development, internal validation, calibration assessment, decision curve analysis, bootstrap resampling, and the random forest comparator analysis were performed in R version 4.3.2. |
| Main R packages | glmne, pROC, rms, rmda, ranger, and boot |
| Analytic cohort | 142 patients with ascertainable primary outcomes. |
| Candidate predictors | 40 prespecified candidate predictors. |
| Primary model | Elastic Net-penalized logistic regression fitted as a binomial logistic model. |
| Cross-validation design | Nested stratified cross-validation. |
| Outer loop | Stratified 10-fold cross-validation for generating out-of-fold predicted probabilities and estimating internal validation performance. |
| Inner loop | Stratified 5-fold cross-validation within each outer-loop training set for hyperparameter tuning. |
| Stratification variable | Binary primary outcome status. |
| Continuous predictors | Median imputation and Z-score standardization. |
| Categorical predictors | Most frequent category imputation and dummy-variable coding. |
| Information leakage prevention | Missing-value imputation, standardization, dummy-variable coding, and model fitting were performed within the training folds only and then applied to the corresponding validation folds. |
| Elastic Net α grid | 0.5, 0.6, 0.7, 0.8, 0.9, and 1.0. |
| Elastic Net λ grid | 1.0, 1.5, 2.0, 2.5, 3.0, 3.5, 4.0, 5.0, 6.0, and 8.0. |
| Hyperparameter selection criterion | Mean AUC in the inner cross-validation was used as the primary criterion; Brier score was used as a secondary criterion when performance was comparable. |
| Final Elastic Net parameters | α=1.0 and λ=6.0. |
| OOF performance of the primary model | AUC=0.820 and Brier score=0.173. |
| OOF calibration metrics | Calibration intercept=-0.018; calibration slope=1.279; calibration-in-the-large=0.026; observed-to-expected ratio=1.009. |
| Bootstrap optimism correction | 1,000 bootstrap resamples were used. Optimism-corrected AUC=0.824, Brier score=0.175, and calibration slope=1.151. |
| Random forest comparator | Implemented using the ranger package and evaluated using the same outer-fold partitions and out-of-fold framework as the Elastic Net model. |
| Model evaluation metrics | AUC, Brier score, calibration metrics, and decision curve analysis. |
| Decision curve analysis | Threshold probabilities were evaluated from 0.05 to 0.80. |

**Table S2. Out-of-fold model performance**

| **Model** | **OOF AUC** | **OOF Brier** | **Youden threshold** | **Accuracy@0.5** | **Sensitivity@0.5** | **Specificity@0.5** | **PPV@0.5** | **Accuracy@Youden** | **Sensitivity@Youden** | **Specificity@Youden** | **PPV@Youden** |
| --- | --- | --- | --- | --- | --- | --- | --- | --- | --- | --- | --- |
| Elastic Net Logistic | 0.820 | 0.173 | 0.622 | 0.725 | 0.768 | 0.667 | 0.759 | 0.775 | 0.683 | 0.900 | 0.903 |
| Random Forest | 0.831 | 0.179 | 0.639 | 0.739 | 0.780 | 0.683 | 0.771 | 0.782 | 0.683 | 0.917 | 0.918 |

**Table S3. Calibration and internal validation metrics for the Elastic Net model**

| **Metric** | **Elastic Net** | **Notes** |
| --- | --- | --- |
| OOF AUC | 0.820 | Outer 10-fold out-of-fold predictions |
| OOF Brier score | 0.173 | Outer 10-fold out-of-fold predictions |
| Calibration intercept | -0.018 | Calibration intercept from OOF predictions |
| Calibration slope | 1.279 | Calibration slope from OOF predictions |
| Calibration-in-the-large | 0.026 | Mean predicted risk compared with observed event rate |
| O/E ratio | 1.009 | Observed-to-expected ratio |
| Bootstrap optimism-corrected AUC | 0.824 | Optimism-corrected internal validation metric |
| Bootstrap optimism-corrected Brier score | 0.175 | Optimism-corrected internal validation metric |
| Bootstrap optimism-corrected calibration slope | 1.151 | Optimism-corrected internal validation metric |

**Table S4. Final Elastic Net coefficients and odds ratios**

| **Variable** | **Coefficient β** | **OR** | **β 2.5%** | **β 97.5%** | **OR 2.5%** | **OR 97.5%** | **Unit** |
| --- | --- | --- | --- | --- | --- | --- | --- |
| (Intercept) | 0.472 | 1.603 |  |  |  |  |  |
| Preoperative pCO₂ | 0.540 | 1.717 | 0.098 | 0.908 | 1.103 | 2.480 | per 1 SD increase |
| LVEF | 0.054 | 1.055 | 0.000 | 0.391 | 1.000 | 1.478 | per 1 SD increase |
| FEV1%pred | -0.913 | 0.401 | -1.341 | -0.221 | 0.261 | 0.802 | per 1 SD increase |
| MVV%pred | -0.061 | 0.941 | -0.696 | 0.000 | 0.499 | 1.000 | per 1 SD increase |
| BSI score | 0.251 | 1.286 | 0.000 | 0.714 | 1.000 | 2.042 | per 1 SD increase |

*Note: Odds ratios are interpreted per 1-SD increase because numerical predictors were standardized in the modeling pipeline. The intercept is shown for model completeness and has no OR confidence interval.*

**Table S5. Variable selection stability across outer folds**

| **Predictor** | **Selection frequency across outer folds** |
| --- | --- |
| FEV1%pred | 100% |
| Preoperative pCO₂ | 100% |
| MVV%pred | 100% |
| BSI score | 90% |
| LVEF | 60% |

**Table S6. Decision-curve net benefit at key threshold probabilities**

| **Threshold probability** | **Elastic Net net benefit** | **Random Forest net benefit** | **Treat-all net benefit** | **Treat-none net benefit** |
| --- | --- | --- | --- | --- |
| 0.20 | 0.479 | 0.467 | 0.472 | 0.000 |
| 0.40 | 0.354 | 0.340 | 0.296 | 0.000 |
| 0.60 | 0.320 | 0.331 | -0.056 | 0.000 |
| 0.62 | 0.325 | 0.317 | -0.112 | 0.000 |

*Note: Across the evaluated threshold probability range of 0.05 to 0.80, the Elastic Net model had greater net benefit than both treat-all and treat-none strategies. The threshold probability around 0.20 was used only as an illustrative clinical decision threshold.*

**Table S7. Classification metrics at illustrative and Youden-derived thresholds**

| **Threshold type** | **Threshold** | **Accuracy** | **Sensitivity** | **Specificity** | **PPV** | **NPV** |
| --- | --- | --- | --- | --- | --- | --- |
| Youden-derived cutoff | 0.622 | 0.775 | 0.683 | 0.900 | 0.903 | 0.675 |
| Illustrative clinical decision threshold | 0.200 | 0.606 | 1.000 | 0.067 | 0.594 | 1.000 |

*Note: The Youden-derived cutoff was used only to summarize classification performance. It should not be interpreted as a mandatory clinical intervention threshold.*

**Table S8. Bootstrap comparison of Random Forest and Elastic Net**

| **Metric** | **Observed difference** | **Bootstrap 95% CI lower** | **Bootstrap 95% CI upper** |
| --- | --- | --- | --- |
| AUC difference (RF - Elastic Net) | 0.011 | -0.027 | 0.051 |
| Brier difference (RF - Elastic Net) | 0.006 | -0.009 | 0.020 |

*Note: Differences are Random Forest minus Elastic Net. Confidence intervals were estimated using 2,000 bootstrap resamples with random seed 42.*

**Table S9. Outer-fold performance of the Elastic Net model**

| **Outer fold** | **Test n** | **Events** | **Event rate** | **AUC** | **Brier** | **Youden threshold** | **Accuracy@Youden** | **Sensitivity@Youden** | **Specificity@Youden** | **PPV@Youden** |
| --- | --- | --- | --- | --- | --- | --- | --- | --- | --- | --- |
| 1 | 15 | 9 | 0.600 | 0.815 | 0.164 | 0.616 | 0.867 | 0.778 | 1.000 | 1.000 |
| 2 | 15 | 9 | 0.600 | 0.889 | 0.157 | 0.699 | 0.867 | 0.889 | 0.833 | 0.889 |
| 3 | 14 | 8 | 0.571 | 0.812 | 0.187 | 0.635 | 0.857 | 0.750 | 1.000 | 1.000 |
| 4 | 14 | 8 | 0.571 | 0.854 | 0.189 | 0.457 | 0.857 | 0.875 | 0.833 | 0.875 |
| 5 | 14 | 8 | 0.571 | 0.854 | 0.171 | 0.675 | 0.857 | 0.875 | 0.833 | 0.875 |
| 6 | 14 | 8 | 0.571 | 0.896 | 0.182 | 0.441 | 0.857 | 1.000 | 0.667 | 0.800 |
| 7 | 14 | 8 | 0.571 | 0.854 | 0.168 | 0.696 | 0.786 | 0.625 | 1.000 | 1.000 |
| 8 | 14 | 8 | 0.571 | 0.771 | 0.209 | 0.650 | 0.714 | 0.500 | 1.000 | 1.000 |
| 9 | 14 | 8 | 0.571 | 0.938 | 0.141 | 0.625 | 0.929 | 1.000 | 0.833 | 0.889 |
| 10 | 14 | 8 | 0.571 | 0.708 | 0.224 | 0.775 | 0.714 | 0.500 | 1.000 | 1.000 |
