# Supplementary material for: An interpretable prognostic model for early unfavorable outcomes after bronchiectasis surgery
Source: BMC Pulm Med. 2026 Jul 16;26:426. doi: 10.1186/s12890-026-04508-4 (PMC13613597; doi:10.1186/s12890-026-04508-4)
Supplement: Supplementary file 1 [file 12890_2026_4508_MOESM1_ESM.zip › Additional file 3.docx]

**Additional file 3: FEV1%pred and MVV%pred Collinearity Analysis**

This additional file summarizes the correlation analysis between FEV1%pred and MVV%pred and the predictor-removal sensitivity analyses used to evaluate the potential impact of collinearity on model robustness.

**Table S1. Correlation diagnostics between FEV1%pred and MVV%pred**

| **Diagnostic** | **Value** |
| --- | --- |
| Pearson correlation | 0.850 |
| Spearman correlation | 0.892 |
| Complete paired observations | 140 |
| Missingness of FEV1%pred | 1.4% |
| Missingness of MVV%pred | 1.4% |

**Table S2. Predictor-removal sensitivity analysis**

| **Model** | **Dropped predictor** | **OOF AUC** | **OOF Brier** | **Youden threshold** | **Accuracy@Youden** | **Sensitivity@Youden** | **Specificity@Youden** | **PPV@Youden** |
| --- | --- | --- | --- | --- | --- | --- | --- | --- |
| Primary full model | None | 0.821 | 0.171 | 0.581 | 0.789 | 0.707 | 0.900 | 0.906 |
| Drop FEV1%pred | FEV1%pred | 0.794 | 0.183 | 0.503 | 0.746 | 0.805 | 0.667 | 0.767 |
| Drop MVV%pred | MVV%pred | 0.822 | 0.170 | 0.562 | 0.796 | 0.720 | 0.900 | 0.908 |

*Note: The sensitivity analysis suggests that FEV1%pred carried more essential predictive information, whereas MVV%pred may provide partially overlapping information.*
