# Supplementary material for: An interpretable prognostic model for early unfavorable outcomes after bronchiectasis surgery
Source: BMC Pulm Med. 2026 Jul 16;26:426. doi: 10.1186/s12890-026-04508-4 (PMC13613597; doi:10.1186/s12890-026-04508-4)
Supplement: Supplementary file 1 [file 12890_2026_4508_MOESM1_ESM.zip › Additional file 4.docx]

**Additional file 4: Exclusion Reasons and Candidate Predictor Missingness**

This additional file summarizes the reasons for exclusion after initial screening and candidate predictor missingness within the final 142-patient analytic cohort.

**Table S1. Reasons for exclusion after initial screening**

| **Reason for exclusion** | **Description** | **n** |
| --- | --- | --- |
| Missing or incomplete Clavien-Dindo grading | Medical records did not provide sufficient information to reliably determine postoperative complication grade | 19 |
| Missing 1-month QoL-B-RSS assessment | Missed scheduled follow-up, unable to complete follow-up assessment, or incomplete QoL-B-RSS questionnaire records | 18 |
| Lost to follow-up | No valid postoperative follow-up information was available to determine the composite outcome | 3 |
| Reoperation before outcome assessment | Reoperation occurred before the scheduled 1-month outcome assessment and interfered with primary outcome ascertainment | 1 |
| Total excluded | Patients excluded because the composite primary outcome could not be reliably determined | 41 |
| Final analytic cohort | Patients with ascertainable composite primary outcome | 142 |
| Initially screened patients | All initially screened bronchiectasis-related lung resection patients | 183 |

*Note: Patients were excluded when the composite primary outcome could not be reliably determined. Reasons were obtained from the screening log, medical record review, and follow-up records.*

**Table S2. Missingness and handling of candidate predictors in the final analytic cohort**

| **Variable** | **Type** | **Missing n** | **Missing %** | **Handling (EN)** |
| --- | --- | --- | --- | --- |
| Intraoperative tracheal intubation-assisted ventilation duration | Continuous | 15 | 10.6% | median imputation + z-score standardization |
| Pulmonary artery diameter (mm) | Continuous | 5 | 3.5% | median imputation + z-score standardization |
| LVEF | Continuous | 5 | 3.5% | median imputation + z-score standardization |
| Preoperative glucose | Continuous | 2 | 1.4% | median imputation + z-score standardization |
| Preoperative AST | Continuous | 2 | 1.4% | median imputation + z-score standardization |
| MVV%pred | Continuous | 2 | 1.4% | median imputation + z-score standardization |
| FEV1/FVC%pred | Continuous | 2 | 1.4% | median imputation + z-score standardization |
| FEV1%pred | Continuous | 2 | 1.4% | median imputation + z-score standardization |
| Preoperative ALT | Continuous | 2 | 1.4% | median imputation + z-score standardization |
| Preoperative albumin | Continuous | 2 | 1.4% | median imputation + z-score standardization |
| Preoperative sodium | Continuous | 2 | 1.4% | median imputation + z-score standardization |
| Preoperative potassium | Continuous | 2 | 1.4% | median imputation + z-score standardization |
| Preoperative blood gas pH | Continuous | 1 | 0.7% | median imputation + z-score standardization |
| High VTE risk (>5) | Categorical | 0 | 0.0% | most-frequent imputation + one-hot encoding (handle_unknown=ignore, drop=first) |
| ASA grade | Categorical | 0 | 0.0% | most-frequent imputation + one-hot encoding (handle_unknown=ignore, drop=first) |
| Complete resection of diseased lesion(s) during surgery | Categorical | 0 | 0.0% | most-frequent imputation + one-hot encoding (handle_unknown=ignore, drop=first) |
| Structural type of bronchiectasis | Categorical | 0 | 0.0% | most-frequent imputation + one-hot encoding (handle_unknown=ignore, drop=first) |
| Intraoperative local pleural changes | Categorical | 0 | 0.0% | most-frequent imputation + one-hot encoding (handle_unknown=ignore, drop=first) |
| Abnormal ECG findings | Categorical | 0 | 0.0% | most-frequent imputation + one-hot encoding (handle_unknown=ignore, drop=first) |
| BSI grade | Categorical | 0 | 0.0% | most-frequent imputation + one-hot encoding (handle_unknown=ignore, drop=first) |
| Thoracoscopic assistance | Categorical | 0 | 0.0% | most-frequent imputation + one-hot encoding (handle_unknown=ignore, drop=first) |
| Number of involved lobes | Continuous | 0 | 0.0% | median imputation + z-score standardization |
| Operative duration (hours) | Continuous | 0 | 0.0% | median imputation + z-score standardization |
| Sex | Categorical | 0 | 0.0% | most-frequent imputation + one-hot encoding (handle_unknown=ignore, drop=first) |
| Other thoracic disease | Categorical | 0 | 0.0% | most-frequent imputation + one-hot encoding (handle_unknown=ignore, drop=first) |
| Preoperative symptoms (hemoptysis or infection) | Categorical | 0 | 0.0% | most-frequent imputation + one-hot encoding (handle_unknown=ignore, drop=first) |
| Age | Continuous | 0 | 0.0% | median imputation + z-score standardization |
| Preoperative creatinine | Continuous | 0 | 0.0% | median imputation + z-score standardization |
| Preoperative pO₂ | Continuous | 0 | 0.0% | median imputation + z-score standardization |
| Preoperative pCO₂ | Continuous | 0 | 0.0% | median imputation + z-score standardization |
| Preoperative hemoglobin (g/L) | Continuous | 0 | 0.0% | median imputation + z-score standardization |
| Preoperative neutrophil percentage | Continuous | 0 | 0.0% | median imputation + z-score standardization |
| History of thoracic surgery | Categorical | 0 | 0.0% | most-frequent imputation + one-hot encoding (handle_unknown=ignore, drop=first) |
| History of hypertension | Categorical | 0 | 0.0% | most-frequent imputation + one-hot encoding (handle_unknown=ignore, drop=first) |
| History of diabetes | Categorical | 0 | 0.0% | most-frequent imputation + one-hot encoding (handle_unknown=ignore, drop=first) |
| History of heart disease | Categorical | 0 | 0.0% | most-frequent imputation + one-hot encoding (handle_unknown=ignore, drop=first) |
| Disease duration (years) | Continuous | 0 | 0.0% | median imputation + z-score standardization |
| Body surface area | Continuous | 0 | 0.0% | median imputation + z-score standardization |
| BMI | Continuous | 0 | 0.0% | median imputation + z-score standardization |
| BSI score | Continuous | 0 | 0.0% | median imputation + z-score standardization |

*Note: Missingness was summarized among the 142 patients included in the final analytic cohort. Imputation procedures were performed within training folds of the cross-validation pipeline.*
