# Supplementary material for: An interpretable prognostic model for early unfavorable outcomes after bronchiectasis surgery
Source: BMC Pulm Med. 2026 Jul 16;26:426. doi: 10.1186/s12890-026-04508-4 (PMC13613597; doi:10.1186/s12890-026-04508-4)
Supplement: Supplementary file 1 [file 12890_2026_4508_MOESM1_ESM.zip › Ethics Approval.pdf]

English annotations for journal submission (non-official translation).

S-4（2.0 版）

新疆医科大学第一附属医院医学伦理委员会审批报告

IRB Approval No.: K202601-65

伦理审批号：K202601-65

研究项目名称<br>Study title	一项可解释的支气管扩张手术早期不良结局预后模型：一项单中心回顾性研究与内部验证		
研究科室<br>Department	胸外科	主要研究者	邓彦超<br>PI
审查文件<br>Documents reviewed	Documents reviewed:<br>1) Ethics review application form<br>2) Study protocol<br>3) Application for waiver/exemption of informed consent<br>4) Declaration of authenticity<br>5) Statement on human genetic resources (as applicable)<br>1. 伦理审查申请表<br>2. 研究方案<br>3. 免除/免签知情同意书申请<br>4. 真实性声明<br>5. 人类遗传资源不涉及出境声明书		
伦理审查方式<br>Type of review	□会议审查　■快速审查	项目来源<br>Project source	研究者自发项目
主审委员<br>Primary reviewer	刘剑		
审查说明<br>Review decision / comments	伦理委员会委员对该研究的相关材料进行了快速审查，认为研究涉及的内容符合伦理原则，同意开展。		
	注：1.本研究跟踪审查频率为 12 个月，请定期向伦理委员会提交跟踪审查报告表。若发生严重不良事件，请及时提交严重不良事件报告。<br>2.本批件有效期为自批准之日起 1 年。<br>3.研究开始前，请申请人完成医学研究登记备案信息系统登记注册。<br>4.如对研究方案或知情同意书进行修改应及时向伦理委员会报告，所涉及的伦理问题均应提交伦理委员会再次审议。<br><br>在研究过程中，研究者应遵守国际《赫尔辛基宣言》及我国四部委联合印发的《涉及人的生命科学和医学研究伦理审查办法》与 GCP 等相关的伦理原则、道德标准及相关的法律、法规、常规、制度等。<br><br>主任或副主任委员签字：[signature]<br>伦理委员会（盖章）<br>Signature (Chair / Vice-chair)<br>日期：2026 年 1 月 22 日<br>[stamp: 新疆医科大学第一附属医院 伦理委员会 6501020451124]<br>Ethics Committee (official seal)		

附件：伦理委员会名单签到及保密协议

FULL ENGLISH TRANSLATION

Approval Report of the Medical Ethics Committee
First Affiliated Hospital of Xinjiang Medical University S-4 (Version 2.0)

**Ethics review / approval No.**	K202601-65
**Study title**	An Interpretable Prognostic Model for Early Unfavorable Outcomes After Bronchiectasis Surgery: A Single-Center Retrospective Cohort Study With Internal Validation
**Department**	Thoracic Surgery
**Principal investigator (PI)**	Yanchao Deng
**Type of ethics review**	Expedited review
**Project source**	Investigator-initiated project
**Primary reviewer**	Jian Liu

Documents reviewed

1 Ethics Review Application Form
2 Study Protocol
3 Application for Waiver/Exemption of Written Informed Consent
4 Statement of Authenticity
5 Statement of Non-involvement of Human Genetic Resources and No Cross-border Transfer

Review decision / comments

Following an expedited review of the submitted materials, the Ethics Committee considered that the study complies with ethical principles and approved the study to proceed.

Notes

1 Continuing review frequency: 12 months. Please submit continuing review/progress reports to the Ethics Committee on schedule. If any serious adverse event occurs, please submit a serious adverse event report promptly.
2 Validity: This approval is valid for 1 year from the date of approval.
3 Before initiation, the applicant shall complete registration in the Medical Research Registration and Filing Information System.
4 Any modification to the study protocol or informed consent documents shall be promptly reported to the Ethics Committee, and ethical issues involved shall be re-submitted for further review.

Compliance statement

Investigators shall comply with the Declaration of Helsinki, the Measures for Ethical Review of Life Science and Medical Research Involving Humans in China, Good Clinical Practice (GCP), and applicable laws, regulations, policies, and institutional requirements.

**Signature of Chairperson / Vice-Chairperson**	For details, please refer to the original file.
**Ethics Committee (official seal)**	For details, please refer to the original file.
**Date**	2026-1-22

Signatures / seal / date (as shown on the original document)

)

Attachments / receipt

Attachment: Sign-in sheet of Ethics Committee members and confidentiality agreement.

Note: This translation is provided for manuscript submission and editorial review only; the original Chinese document remains the official version.
