# Supplementary material for: An interpretable prognostic model for early unfavorable outcomes after bronchiectasis surgery
Source: BMC Pulm Med. 2026 Jul 16;26:426. doi: 10.1186/s12890-026-04508-4 (PMC13613597; doi:10.1186/s12890-026-04508-4)
Supplement: Supplementary file 1 [file 12890_2026_4508_MOESM1_ESM.zip › Table S1.docx]

| model | AUC | Brier |
| --- | --- | --- |
| Primary model | 0.820528455 | 0.171091797 |
| Model excluding FEV1%pred | 0.793699187 | 0.182870266 |
| Model excluding MVV%pred | 0.82195122 | 0.170293468 |

Table S1 Sensitivity analysis for the inclusion of FEV1%pred and MVV%pred in the primary model

The primary model was the final Elastic Net model. Alternative models were reconstructed after excluding FEV1%pred or MVV%pred separately, with all other analytic settings unchanged. Excluding FEV1%pred worsened model performance (AUC 0.821 to 0.794; Brier score 0.171 to 0.183), whereas excluding MVV%pred produced minimal change (AUC 0.822; Brier score 0.170). This suggests that FEV1%pred carried more key predictive information, while MVV%pred added relatively limited incremental value.
